# Supplementary figures and images for: Association of serum fetuin-B with insulin resistance and pre-diabetes in young Chinese women: evidence from a cross-sectional study and effect of liraglutide
Source: PeerJ. 2021 Aug 20;9:e11869. doi: 10.7717/peerj.11869 (PMC8381879; doi:10.7717/peerj.11869)

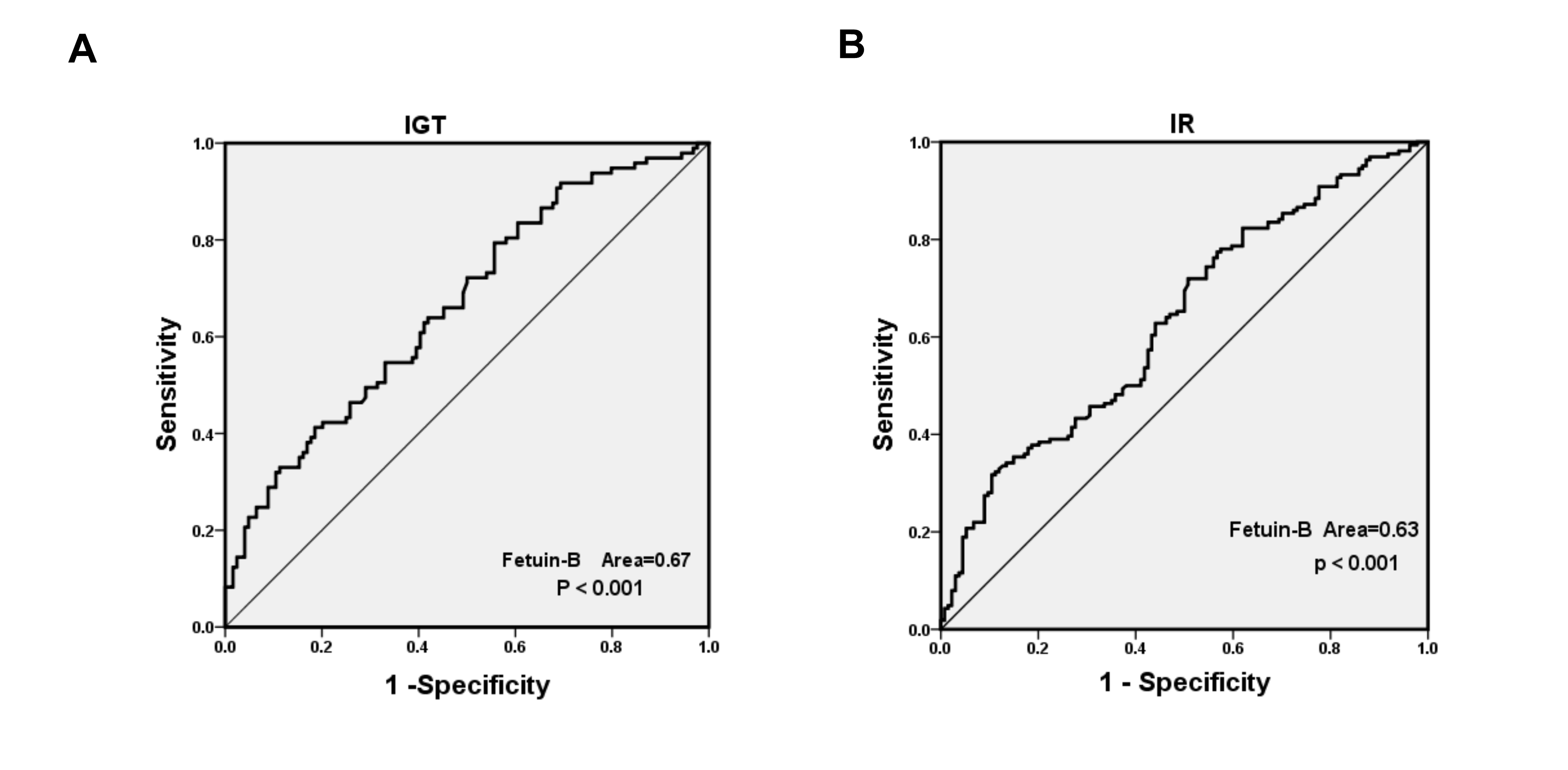

Supplement: Supplemental Information 2 [file peerj-09-11869-s002.png]
