# Supplementary material for: Association of serum fetuin-B with insulin resistance and pre-diabetes in young Chinese women: evidence from a cross-sectional study and effect of liraglutide
Source: PeerJ. 2021 Aug 20;9:e11869. doi: 10.7717/peerj.11869 (PMC8381879; doi:10.7717/peerj.11869)
Supplement: Supplemental Information 3 [file peerj-09-11869-s003.docx]

**Supplemental Table S1** Association of circulating Fetuin-B levels with IR in fully adjusted models.

|  | IR | | |
| --- | --- | --- | --- |
| Model adjust | OR | 95%CI | p |
| Age | 1.146 | 1.075-1.221 | < 0.001 |
| Age, BP | 1.132 | 1.059-1.211 | < 0.001 |
| Age, BP, TG | 1.105 | 1.030-1.185 | < 0.01 |
| Age, BP, TG, HbA1c | 1.086 | 1.010-1.169 | < 0.05 |

Results of multivariate logistic regression analysis were presented as the odds ratio (OR) of being in IR status increase in serum Fetuin-B levels.
