# Supplementary material for: Association of serum fetuin-B with insulin resistance and pre-diabetes in young Chinese women: evidence from a cross-sectional study and effect of liraglutide
Source: PeerJ. 2021 Aug 20;9:e11869. doi: 10.7717/peerj.11869 (PMC8381879; doi:10.7717/peerj.11869)
Supplement: Supplemental Information 4 [file peerj-09-11869-s004.docx]

**Supplementary Table S2** Association of circulating Fetuin-B levels with IGT in fully adjusted models.

|  | IGT | | |
| --- | --- | --- | --- |
| Model adjust | OR | 95%CI | p |
| Age | 1.185 | 1.097-1.280 | < 0.001 |
| Age, FAT% | 1.175 | 1.057-1.307 | < 0.01 |
| Age, FAT%, BMI | 1.173 | 1.055-1.305 | < 0.01 |
| Age, FAT%, BMI, WC | 1.148 | 1.030-1.279 | < 0.05 |
| Age, FAT%, BMI, WC, BP | 1.141 | 1.022-1.274 | < 0.05 |
| Age, FAT%, BMI, WC, BP, TG | 1.129 | 1.011-1.260 | < 0.05 |

Results of multivariate logistic regression analysis were presented as the odds ratio (OR) of being in IGT status increase in serum Fetuin-B levels.
