# Supplementary material for: Association of serum fetuin-B with insulin resistance and pre-diabetes in young Chinese women: evidence from a cross-sectional study and effect of liraglutide
Source: PeerJ. 2021 Aug 20;9:e11869. doi: 10.7717/peerj.11869 (PMC8381879; doi:10.7717/peerj.11869)
Supplement: Supplemental Information 7 [file peerj-09-11869-s007.doc]

**Ling Li**

**Clinical Research Protocol**

**Association of serum Fetuin-B with insulin resistance and pre-diabetes in young Chinese women: evidence from a cross-sectional study and effect of liraglutide**

| Protocol Number: | ChiCTR-OCC-11001422 |
| --- | --- |
| Version Date: | 2014.12.1 |
| Investigational Product: | Liraglutide |
| NDA Number: | 22341 S-027 |
| Development Phase: | Phase IV clinical study |
| Sponsor: | Ling Li  Chongqing Medical University  Chongqing, China |
| Funding Organization: | The National Natural Science Foundation of China (No. 81670755), the Natural Science Foundation of Chongqing (No. cstc2017jcyjAX0050) |
| Principal Investigator: | Name: Xuyun Xia  Telephone: (+86) 13368379720  Fax: no  E-mail: Jennyxia123@163.com |
| Medical Monitor: | Name: Shiyao Xue  Telephone: (+86) 19942331709  Fax: no  E-mail: 1098499048@qq.com |
| Coordinating Center: | the Second Affiliated Hospital of Chongqing Medical University |

| **Approval:** | | |
| --- | --- | --- |
| Ling Li (Professor) |  | 2014.9.1 |
| **This confidential information about an investigational product is provided for the exclusive use of investigators of this product and is subject to recall at any time. The information in this document may not be disclosed unless federal or state law or regulations require such disclosure. Subject to the foregoing, this information may be disclosed only to those persons involved in the study who have a need to know, with the obligation not to further disseminate this information.** | | |

**PROTOCOL AGREEMENT**

I have read the protocol specified below. In my formal capacity as Investigator, my duties include ensuring the safety of the study subjects enrolled under my supervision and providing [Ling Li] with complete and timely information, as outlined in the protocol. It is understood that all information pertaining to the study will be held strictly confidential and that this confidentiality requirement applies to all study staff at this site. Furthermore, on behalf of the study staff and myself, I agree to maintain the procedures required to carry out the study in accordance with accepted GCP principles and to abide by the terms of this protocol.

Protocol Number: ChiCTR-OCC-11001422

Protocol Title: Association of serum Fetuin-B with insulin resistance and pre-diabetes in young Chinese women: evidence from a cross-sectional study and effect of liraglutide

Protocol Date: 2014.12.1

|  | | |  |  |
| --- | --- | --- | --- | --- |
| Investigator Signature: Xuyun Xia | | |  | 2014.12.1 |
|  | | | | |
| Print Name and Title | | | | |
| Site # | Chongqing |  | | |
| Site Name | Chongqing Medical University | | | |
| Address | No. 1, Medical School Road, Yuzhong District, Chongqing | | | |
|  |  | | | |
|  |  | | | |
|  |  | | | |
| Phone Number | 023 6848 6151 | | | |

**TABLE OF CONTENTS**

[1 BACKGROUND 4](#__RefHeading___Toc262733374)

1.1 Overview of Non-Clinical Studies [9](#__RefHeading___Toc262733375)

[1.2 Overview of Clinical Studies 9](#__RefHeading___Toc262733376)

[2 STUDY RATIONALE 9](#__RefHeading___Toc262733377)

[2.1 Risk / Benefit Assessment 9](#__RefHeading___Toc262733378)

[3 STUDY OBJECTIVES 9](#__RefHeading___Toc262733379)

[3.1 Primary Objective 9](#__RefHeading___Toc262733380)

[3.2 Secondary Objectives 9](#__RefHeading___Toc262733381)

[4 STUDY DESIGN 9](#__RefHeading___Toc262733382)

[4.1 Study Overview 9](#__RefHeading___Toc262733383)

[5 Criteria for evaluation 10](#__RefHeading___Toc262733384)

[5.1 Primary Efficacy Endpoint 10](#__RefHeading___Toc262733385)

[5.2 Secondary Efficacy Endpoints 10](#__RefHeading___Toc262733386)

[5.3 Safety Evaluations 10](#__RefHeading___Toc262733387)

[5.4 Other Evaluations 10](#__RefHeading___Toc262733388)

[6 SUBJECT SELECTION 10](#__RefHeading___Toc262733389)

[6.1 Study Population 10](#__RefHeading___Toc262733390)

[6.2 Inclusion Criteria 10](#__RefHeading___Toc262733391)

[6.3 Exclusion Criteria 11](#__RefHeading___Toc262733392)

[7 Concurrent Medications 11](#__RefHeading___Toc262733393)

[7.1 Allowed 11](#__RefHeading___Toc262733394)

[7.2 Prohibited 11](#__RefHeading___Toc262733395)

[8 STUDY TREATMENTS 11](#__RefHeading___Toc262733396)

[8.1 Method of Assigning Subjects to Treatment Groups 11](#__RefHeading___Toc262733397)

[8.3 Test Formulation 12](#__RefHeading___Toc262733399)

[8.4 Supply of Study Medication at the Site 13](#__RefHeading___Toc262733400)

[8.5 Study Medication Accountability 14](#__RefHeading___Toc262733401)

[8.6 Measures of Treatment Compliance 14](#__RefHeading___Toc262733402)

[9 STUDY PROCEDURES AND GUIDELINES 14](#__RefHeading___Toc262733403)

[9.1 Clinical Assessments 14](#__RefHeading___Toc262733404)

[9.2 Clinical Laboratory Measurements 15](#__RefHeading___Toc262733405)

[9.3 Pharmacokinetic Measurements 16](#__RefHeading___Toc262733406)

[9.4 Research Laboratory Measurements 16](#__RefHeading___Toc262733407)

[10 EVALUATIONS BY VISIT 16](#__RefHeading___Toc262733408)

[10.1 Visit 1 16](#__RefHeading___Toc262733409)

[10.2 Visit 2 17](#__RefHeading___Toc262733410)

[10.3 Visit 3 17](#__RefHeading___Toc262733411)

[10.6 Early Withdrawal Visit 18](#__RefHeading___Toc262733414)

[11 ADVERSE Experience REPORTING AND DOCUMENTATION 18](#__RefHeading___Toc262733415)

[11.1 Adverse Events 18](#__RefHeading___Toc262733416)

[11.2 Serious Adverse Experiences (SAE) 20](#__RefHeading___Toc262733417)

[11.3 Protocol Defined Important Medical Findings Requiring Real Time Reporting 20](#__RefHeading___Toc262733418)

[11.4 Medical Monitoring 20](#__RefHeading___Toc262733419)

[11.5 Safety Management Plan](#__RefHeading___Toc262733420) 4

[12 DISCONTINUATION And Replacement of subjects 20](#__RefHeading___Toc262733421)

[12.1 Withdrawal of Subjects 20](#__RefHeading___Toc262733422)

[12.3 Replacement of Subjects 21](#__RefHeading___Toc262733423)

[13 PRotocol Violations 22](#__RefHeading___Toc262733424)

[14 DATA SAFETY MONITORING (optional Section – Include when appropriate) 22](#__RefHeading___Toc262733425)

[15 STATISTICAL METHODS AND CONSIDERATIONS 22](#__RefHeading___Toc262733426)

[15.1 Data Sets Analyzed 22](#__RefHeading___Toc262733427)

[15.2 Demographic and Baseline Characteristics 22](#__RefHeading___Toc262733428)

[15.3 Analysis of Primary Endpoint 23](#__RefHeading___Toc262733429)

[15.4 Analysis of Secondary Endpoints 23](#__RefHeading___Toc262733430)

[15.5 Interim Analysis 23](#__RefHeading___Toc262733431)

[15.6 Sample Size 23](#__RefHeading___Toc262733432)

[16 DATA COLLECTION, RETENTION AND MONITORING 23](#__RefHeading___Toc262733433)

[16.1 Data Collection Instruments 23](#__RefHeading___Toc262733434)

[16.2 Data Management Procedures 24](#__RefHeading___Toc262733435)

[16.3 Data Quality Control and Reporting 24](#__RefHeading___Toc262733436)

[16.4 Archival of Data 24](#__RefHeading___Toc262733437)

[16.5 Availability and Retention of Investigational Records 24](#__RefHeading___Toc262733438)

[16.6 Monitoring 25](#__RefHeading___Toc262733439)

[16.7 Subject Confidentiality 25](#__RefHeading___Toc262733440)

[17 ADMINISTRATIVE, ETHICAL, REGULATORY CONSIDERATIONS 25](#__RefHeading___Toc262733441)

[17.1 Protocol Amendments 25](#__RefHeading___Toc262733442)

[17.2 Institutional Review Boards and Independent Ethics Committees 25](#__RefHeading___Toc262733443)

[17.3 Informed Consent Form 26](#__RefHeading___Toc262733444)

[17.4 Publications 26](#__RefHeading___Toc262733445)

[17.5 Investigator Responsibilities 27](#__RefHeading___Toc262733446)

List of Abbreviations

| **AE** | adverse event |
| --- | --- |
| **ADA** | American Diabetes Association |
| **BMI** | body mass index |
| **CRF** | case report form |
| **CSE** | Chinese Society of Endocrinology |
| **DMC** | Data Monitoring Committee |
| **DSMB** | Data Safety Monitoring Board |
| **EHC** | Euglycemic- hyperinsulinemic clamp |
| **ELISA** | enzyme-linked immunosorbent assay |
| **FDA** | Food and Drug Administration |
| **Fins** | fasting insulin |
| **GCP** | Good Clinical Practice |
| **HIPAA** | Health Insurance Portability and Accountability Act of 1996 |
| **HOMA2-IR** | homeostasis model assessment of insulin resistance |
| **ICF** | informed consent form |
| **ICH** | International Conference on Harmonisation |
| **IEC** | Independent Ethics Committee |
| **IGT** | impaired glucose tolerance |
| **IR** | insulin resistance |
| **IRB** | Institutional Review Board |
| **Lira** | liraglutide |
| **LSD** | Fisher’s Least Significant Difference |
| **OGTT** | oral glucose tolerance test |
| **PI** | Principal Investigator |
| **SAE** | serious adverse experience |
| **WHR** | waist-to-hip ratio |
| **2h-BG** | 2-hour blood glucose post-glucose load |

Protocol Synopsis

| **TITLE** | Association of serum Fetuin-B with insulin resistance and pre-diabetes in young Chinese women: evidence from a cross-sectional study and effect of liraglutide |
| --- | --- |
|  |  |
| **SPONSOR** | Ling Li |
|  |  |
| **FUNDING ORGANIZATION** | The National Natural Science Foundation of China (No. 81670755), the Natural Science Foundation of Chongqing (No.cstc2017jcyjAX0050) |
| **NUMBER OF SITES** | +86, +023 |
|  |  |
| **RATIONALE** | Fetuin-B has been reported to be involved in glucose and lipid metabolism and associated with the occurrence of diabetes. The main purpose of this study is to explore the changes of circulating Fetuin-B in young women with pre-diabetes and to analyze the relationship between Fetuin-B and the occurrence and development of IR.. |
|  |  |
| **STUDY DESIGN** | This is a cross-sectional study. |
|  |  |
| **PRIMARY OBJECTIVE** | Explore the changes of circulating Fetuin-B in young women with pre-diabetes and to analyze the relationship between Fetuin-B and the occurrence and development of IR |
|  |  |
| **SECONDARY OBJECTIVES** | Explore the effects of liraglutide (Lira) on Fetuin-B and obesity. |
|  |  |
| **NUMBER OF SUBJECTS** | 304 |
|  |  |
| **SUBJECT SELECTION**  **CRITERIA** | Inclusion Criteria:  IGT was diagnosed according to the American Diabetes Association (ADA)(American Diabetes 2012). The presence of pre-diabetes was defined by the occurrence of IGT at 2h-OGTT. M-value < 6.28 was considered to have IR.  Lira: included age of 18-35 and BMI of 25-35 kg/m^2^  Exclusion Criteria:  type 1 diabetes mellitus (T1DM), T2DM, hypertension, and other important organ diseases such as heart, liver, and kidney  Lira: included the family history of thyroid tumor, the history of medullary thyroid carcinoma, severe gastrointestinal disease, acute pancreatitis, and pregnancy. |
|  |  |
| **TEST PRODUCT, DOSE, AND ROUTE OF ADMINISTRATION** | Product Lira at 1.8mg dose  Liar was injected subcutaneously once a day. The dose increased from 0.6 mg to 1.8 mg |
|  |  |
|  |  |
| dURATION OF SUBJECT PARTICIPATION AND DURATION OF STUDY | Subjects will be on study for up to 24 weeks  **Screening :**  2 weeks  **Treatment:** 24 weeks  **Follow-up:** 24 weeks  The total duration of the study is expected to be 24weeks. |
| **E** |  |
| **CONCOMMITANT MEDICATIONS** | No |
|  |  |
| **Efficacy Evaluations** | Physical examination and biochemical tests, OGTT, and EHC tests were performed before treatment, 12 and 24 weeks after treatment. |
| ***Primary endpoint*** | - 24 weeks after application of lira |
| ***Secondary endpoints*** | - Serious adverse events within 24 weeks |
| **Other Evaluations** | BMI, Fat %, WC, TG, FBG, FIns, HbA1c and HOMA2-IR |
| **Safety Evaluations** | Change in clinical safety labs from baseline to 24weeks  Incidence of adverse events is 0% |
| **Planned Interim Analyses** | When approximately 50% of patients have completed the study through outpatient department of Endocrinology, an interim analysis for safety will be conducted by an independent data monitoring committee. Serious adverse events will be monitored by the committee on an ongoing basis throughout the study. |
|  |  |
| **STATISTICS**  **Primary Analysis Plan** | SPSS version 24.0 software was used for statistical analysis. Data were expressed as mean±SD or median. Variables that are not normally distributed were converted logarithmically. Analysis of variance was used for comparison among groups, and LSD test was used for comparison between two groups. The relationship between serum Fetuin-B and other variables was evaluated by linear regression analysis with controlled covariates. Multiple linear regression analysis was used to identify independent factors associated with serum Fetuin-B. Multiple logistic regression analysis was conducted to understand the association of serum Fetuin-B with IR and IGT. Cochran-Armitage trend test and Row Mean Scores analysis were performed to investigate the changing trend of the Fetuin-B level in IGT and IR-NG groups. ROC curves were constructed to evaluate the sensitivity and specificity of serum Fetuin-B in predicting IR and IGT |
| **Rationale for Number of Subjects** | A total of 304 young women were enrolled in this study, including 80 newly diagnosed IR patients (IR-NG group), 97 individuals with impaired glucose tolerance (IGT group), and 127 healthy women (NGT group). The sample size is sufficient, the distribution of each group is even and appropriate, the setting of lira subgroups is reasonable, which make the results convincing. |

# BACKGROUND

T2DM has been considered a chronic disease related to the elderly, but with rapid economic and social development in recent years, changes in lifestyle and diet structure have resulted in increasing rates of T2DM and obesity in adolescents. Serum Fetuin-B is considered to be an adipo-hepatokine and a member of the cystatin superfamily of cysteine protease inhibitors, and serum Fetuin-B levels are significantly related to IR. Therefore, the relationship between serum Fetuin-B and IR is still controversial.

## Overview of Non-Clinical Studies

The results from Meex et al. showed that Fetuin-B could induce IGT by inhibiting the effect of insulin on myotubes and hepatocytes in mice. In obese mice, knockout of the Fetuin-B gene significantly improved impaired glucose tolerance (IGT). In an in vivo study, it was shown that Fetuin-B may regulate blood glucose through a non-insulin signaling pathway, but the specific mechanism is still unclear.

## Overview of Clinical Studies

In a clinical study, Qu et al. found that the serum Fetuin-B levels in T2DM patients were significantly higher than those in IGT and normal subjects, and serum Fetuin-B was correlated with TG, fasting insulin, homeostasis model assessment of insulin resistance (HOMA-IR), and insulin secretion in the first phase. In addition, it has been reported that serum Fetuin-B levels are significantly increased in patients with nonalcoholic fatty liver disease (NAFLD), and related to IR. In contrast, a recent study showed that the mRNA expression of Fetuin-B in the liver was not associated with HOMA-IR. For more detail refer to the Investigator’s Brochure.

# STUDY RATIONALE

There have been some reports that Fetuin-B is related to metabolic diseases in recent years. However, most of the previous studies were cross-sectional and descriptive studies. They were merely a preliminary exploration of Fetuin-B, and there was no intervention experiment to fully prove the relationship between Fetuin-B and human IR, and no state-of-the-art methodology was used. In addition, there have been many confounding factors in previous studies, such as middle-aged and elderly subjects, most of whom had long-term dyslipidemia or other metabolic diseases, and with a long history of drug treatment. In addition, age and gender also have important effects on obesity and IR. Therefore, if applied to clinical work, the previous results have some limitations.

# STUDY OBJECTIVES

## Primary Objective

The primary objective is to assess the association of serum Fetuin-B with IR-NG and IGT.

## Secondary Objectives

The secondary objective is to assess the clinical efficacy as measured by the change in physical examination and biochemical tests, OGTT, and EHC tests before treatment, 12 and 24 weeks after treatment.

# STUDY DESIGN

## Study Overview

This is a cross-sectional study. 304 of subjects are enrolled and applied with biochemical tests, OGTT, and EHC tests on admission. In the subgroup, each subject will be administered lira at the dose of 1.8mg, once a day, and evaluations will be taken before treatment, 12 and 24 weeks after treatment.

Screening data will be reviewed to determine subject eligibility. Subjects who meet all inclusion criteria and none of the exclusion criteria will be entered into the study.

The following treatment regimens will be used:

- Experimental treatment Lira at the following doses: increased from 0.6 mg to 1.8 mg

Total duration of the study is expected to be 24 weeks.

# Criteria for evaluation

## Primary Efficacy Endpoint

24 week after application of lira (It has been well known that Lira is generally used at doses up to 1.8 mg as an anti-diabetic agent, but higher doses (up to 3.0 mg) and long terms have been approved for weight loss in the US and Europe in obese individuals, irrespective of hyperglycemia. Given the weight characteristics of the Chinese population, we treated IR patients with 1.8 mg Lira for 24 weeks).

## Secondary Efficacy Endpoints

- Serious adverse events within 24 weeks.

## Safety Evaluations

- The basic indicators of the patient are all in the normal range.
- Incidence of adverse events is 0%.

# SUBJECT SELECTION

## Study Population

Subjects with a diagnosis of IGT and IR who meet the inclusion and exclusion criteria will be eligible for participation in this study.

## Inclusion Criteria

1. Female 18-35 years of age at Visit outpatient.
2. Documentation of IGT/IR diagnosis as evidenced by one or more clinical features consistent with the IGT/IR phenotype and one or more of the following criteria:

- IGT：2-h PG in the 75-g OGTT 140 mg/dl (7.8mmol/l) to 199 mg/dl (11.0 mmol/l)
- M-value < 6.28 was considered to have IR

1. Written informed consent (and assent when applicable) obtained from subject or subject’s legal representative and ability for subject to comply with the requirements of the study.

## Exclusion Criteria

1. Pregnant, breastfeeding, or unwilling to practice birth control during participation in the study.
2. Presence of a condition or abnormality that in the opinion of the Investigator would compromise the safety of the patient or the quality of the data.
3. type 1 diabetes mellitus (T1DM), T2DM, hypertension, and other important organ diseases such as heart, liver, and kidney.
4. family history of thyroid tumor, the history of medullary thyroid carcinoma, severe gastrointestinal disease, acute pancreatitis

# Concurrent Medications

All subjects should be maintained on the same medications throughout the entire study period, as medically feasible, with no introduction of new chronic therapies.

## Allowed Medications and Treatments

Standard therapy for lira is allowed except for treatments noted in the exclusion criteria described above.Those subjects did not use any drugs in the last 3 months.

# STUDY TREATMENTS

## Method of Assigning Subjects to Treatment Groups

A total of 26 overweight / obese women with IR participated in GLP-1RA intervention for 24 weeks. Included age of 18-35 and BMI of 25-35 kg/m2.

## Formulation of Test Product

Lira is developed by Novo Nordisk, high doses (up to 3.0mg) of lira is for subcutaneously administration in the management of overweight/obese patients. Lira is a colorless or almost colorless clear isotonic liquid, pH=8.15.

## Supply of Study Drug at the Site

The designee will ship Lira to the investigational sites. The initial study drug shipment will be shipped after site activation (all required regulatory documentation has been received by the Sponsor and a contract has been executed). Subsequent study drug shipments will be made after site request for resupply.

### Dosage/Dosage Regimen

Liar was injected subcutaneously once a day. The dose increased from 0.6 mg to 1.8 mg. Inclusion criteria included age of 18-35 and BMI of 25-35 kg/m2.

### Dispensing

Lira was dispensed by pharmacist and Injected by investigator.

### Administration Instructions

Liar was injected subcutaneously once a day at the dose from 0.6 mg to 1.8 mg. The timing of the dose adjustment is determined by the investigator.

## Supply of Study Drug at the Site

The subjects were enrolled for intervention with lira due to ovarian dysfunction and refractory menstrual problems.All subjects signed informed consent before treatment. 26 subjects were followed up to the end of the study. Lira was supplied daily by the Pharmacy Department and delivered to the day clinic.

### Storage

Study drug should be stored by the study site in the refrigerator (2 to 8ºC) and and protected from light. If the temperature of study drug storage in the clinic/pharmacy exceeds or falls below this range, this should be reported to the Sponsor or designee and captured as a deviation.

## Study Drug Accountability

An accurate and current accounting of the dispensing and return of study drug for each subject will be maintained on an ongoing basis by a member of the study site staff. The number of study drug dispensed and returned by the subject will be recorded on the Investigational Drug Accountability Record. The study monitor will verify these documents throughout the course of the study.

## Measures of Treatment Compliance

Subjects will be asked to come to the study site and be injected lira at the same time every day. If they did not come, the designee would call to remind them to ensure their compliance.

# STUDY PROCEDURES AND GUIDELINES

A Schedule of Events representing the required testing procedures to be performed for the duration of the study is diagrammed in Appendix 1.

Prior to conducting any study-related activities, written informed consent and the Health Insurance Portability and Accountability Act (HIPAA) authorization must be signed and dated by the subject or subject’s legal representative. If appropriate, assent must also be obtained prior to conducting any study-related activities.

## Clinical Assessments

### Concomitant Medications

All concomitant medication and concurrent therapies will be documented at Baseline/Screening, 12 and 24 weeks after treatment. Dose, route, unit frequency of administration, and indication for administration and dates of medication will be captured.

### Demographics

Demographic information (date of birth, gender, race) will be recorded at Screening.

### Medical History

Relevant medical history, including history of current disease, other pertinent respiratory history, and information regarding underlying diseases will be recorded at Screening.

### Physical Examination

After fasting for 10-12 h, all subjects were given a physical examination and biochemical tests by professionals. The physical examination included body weight, blood pressure (BP), waist circumference (WC), and body fat content (Fat %). A complete physical examination will be performed by either the investigator or a subinvestigator who is a physician before treatment. Qualified staff (MD, NP, RN, and PA) may complete the abbreviated physical exam at all other visits. New abnormal physical exam findings must be documented and will be followed by a physician or other qualified staff at the next scheduled visit.

### Vital Signs

Physical examination and biochemical tests, OGTT, and EHC tests were performed before treatment, 12 and 24 weeks after treatment.

### Adverse Events

Information regarding occurrence of adverse events will be captured throughout the study. Duration (start and stop dates and times), severity/grade, outcome, treatment and relation to study drug will be recorded on the case report form.

## Clinical Laboratory Measurements

### Serum Fetuin-B concentration test

Serum Fetuin-B concentration was determined by an ELISA kit (RayBiotech, Inc. Norcross, GA, USA). The detection limit for Fetuin-B was 4.0 ng/ml. Intra- and inter-assay variations (CV) were 10 % and 12 %, respectively.

### Biochemical parameters

Blood will be obtained and sent to each site’s clinical chemistry lab for determination of total cholesterol (TC), triglyceride (TG), high-density lipoprotein cholesterol (HDL-C), low-density lipoprotein cholesterol (LDL-C), free fatty acid (FFA), HbA1c, FBG, 2-hour postprandial blood glucose (2h-BG), fasting insulin (FIns), and 2-hour postprandial insulin (2h- INS).

### OGTT Test

After 10-12 hours of fasting overnight, all subjects were given 75g glucose orally and the OGTT test was performed at 8:00 AM. During the OGTT, blood samples were collected for the measurements of blood glucose, insulin, and serum Fetuin-B at 0, 30, 60, and 120 min.

### EHC Test

The EHC was performed in all subjects. During the EHC, human insulin (1mU/kg/min) was continuously infused for 2 hours. At the same time, the infusion rate of the 20% glucose was adjusted according to the blood glucose levels, tested every 15 min, to maintain blood glucose at the basic level (4.5-5.5 mmol/L).

# EVALUATIONS BY VISIT

## Visit 1 (0 Week)

1. Review the study with the subject (subject’s legal representative) and obtain written informed consent and approved by the ethics committee of the Second Affiliated Hospital of Chongqing Medical University .
2. Assign the subject a unique screening number.
3. Record demographics data.
4. Record medical history,, and prior treatments.
5. Record concomitant medications.
6. Perform a complete physical examination.
7. Perform and record vital signs.
8. Perform and record results of blood pressure testing.
9. Collect blood for clinical laboratory tests (TC, TG, HDL-C, LDL-C, FFA, HbA1c, FBG, 2h-BG, FIns, 2h- INS).
10. Schedule subject for Visit 2 after 12 weeks.
11. List all additional procedures, such as Randomize subject, Dispense study drug, Initiate subject diary, etc.

## Visit 2 (12 weeks)

1. Record any Adverse Experiences and/or Review subject diary for adverse experiences and dosing compliance.
2. Concomitant medications review.
3. Perform abbreviated physical examination.
4. Perform and record vital signs.
5. Collect blood for clinical laboratory tests (TC, TG, HDL-C, LDL-C, FFA, HbA1c, FBG, 2h-BG, FIns, 2h- INS).
6. List all additional procedures.

## Visit 3 (24 weeks)

1. Record any Adverse Experiences and/or Review subject diary for adverse experiences and dosing compliance.
2. Record changes to concomitant medications.
3. Perform abbreviated physical examination.
4. Perform and record vital signs.
5. Collect blood for clinical laboratory tests (TC, TG, HDL-C, LDL-C, FFA, HbA1c, FBG, 2h-BG, FIns, 2h- INS).
6. List all additional procedures.

## Early Withdrawal Visit

1. Record any Adverse Experiences and/or Review subject diary for adverse experiences and exclusionary medication use.
2. Record changes to concomitant medications.
3. Perform complete physical examination.
4. Perform and record vital signs.
5. Collect blood for clinical laboratory tests (TC, TG, HDL-C, LDL-C, FFA, HbA1c, FBG, 2h-BG, FIns, 2h- INS).

# ADVERSE Experience REPORTING AND DOCUMENTATION

## Adverse Events

An adverse event (AE) is any untoward medical occurrence in a clinical investigation of a patient administered a pharmaceutical product and that does not necessarily have a causal relationship with the treatment. An AE is therefore any unfavorable and unintended sign (including an abnormal laboratory finding), symptom or disease temporally associated with the administration of an investigational product, whether or not related to that investigational product. An unexpected AE is one of a type not identified in nature, severity, or frequency in the current Investigator’s Brochure or of greater severity or frequency than expected based on the information in the Investigator’s Brochure.

The Investigator will probe, via discussion with the subject, for the occurrence of AEs during each subject visit and record the information in the site’s source documents. Adverse events will be recorded in the patient CRF. Adverse events will be described by duration (start and stop dates and times), severity, outcome, treatment and relation to study drug, or if unrelated, the cause.

**AE Severity**

The National Cancer Institute’s Common Terminology Criteria for Adverse Events (CTCAE) Version 3.0 should be used to assess and grade AE severity, including laboratory abnormalities judged to be clinically significant. The modified criteria can be found in the study manual. If the experience is not covered in the modified criteria, the guidelines shown in Table 1 below should be used to grade severity. It should be pointed out that the term “severe” is a measure of intensity and that a severe AE is not necessarily serious.

Table 1. AE Severity Grading

| **Severity (Toxicity Grade)** | **Description** |
| --- | --- |
| Mild (1) | Transient or mild discomfort; no limitation in activity; no medical intervention or therapy required. The subject may be aware of the sign or symptom but tolerates it reasonably well. |
| Moderate (2) | Mild to moderate limitation in activity, no or minimal medical intervention/therapy required. |
| Severe (3) | Marked limitation in activity, medical intervention/therapy required, hospitalizations possible. |
| Life-threatening (4) | The subject is at risk of death due to the adverse experience as it occurred. This does not refer to an experience that hypothetically might have caused death if it were more severe. |

**AE Relationship to Study Drug**

The relationship of an AE to the study drug should be assessed using the following the guidelines in Table 2.

Table 2. AE Relationship to Study Drug

| **Relationship to Drug** | **Comment** |
| --- | --- |
| Definitely | Previously known toxicity of agent; or an event that follows a reasonable temporal sequence from administration of the drug; that follows a known or expected response pattern to the suspected drug; that is confirmed by stopping or reducing the dosage of the drug; and that is not explained by any other reasonable hypothesis. |
| Probably | An event that follows a reasonable temporal sequence from administration of the drug; that follows a known or expected response pattern to the suspected drug; that is confirmed by stopping or reducing the dosage of the drug; and that is unlikely to be explained by the known characteristics of the subject’s clinical state or by other interventions. |
| Possibly | An event that follows a reasonable temporal sequence from administration of the drug; that follows a known or expected response pattern to that suspected drug; but that could readily have been produced by a number of other factors. |
| Unrelated | An event that can be determined with certainty to have no relationship to the study drug. |

## Serious Adverse Experiences (SAE)

An SAE is defined as any AE occurring at any dose that results in any of the following outcomes:

- death
- a life-threatening adverse experience
- inpatient hospitalization or prolongation of existing hospitalization
- a persistent or significant disability/incapacity
- a congenital anomaly/birth defect

Other important medical events may also be considered an SAE when, based on appropriate medical judgment, they jeopardize the subject or require intervention to prevent one of the outcomes listed.

### Serious Adverse Experience Reporting

Study sites will document all SAEs that occur (whether or not related to study drug) per [UCSF CHR Guidelines](http://www.research.ucsf.edu/chr/Guide/Adverse_Events_Guidelines.asp#2). The collection period for all SAEs will begin after informed consent is obtained and end after procedures for the final study visit have been completed.

In accordance with the standard operating procedures and policies of the local Institutional Review Board (IRB)/Independent Ethics Committee (IEC), the site investigator will report SAEs to the IRB/IEC.

## Medical Monitoring

Shiyao Xue should be contacted directly at these numbers to report medical concerns or questions regarding safety.

Phone: (+86) 19942331709

# DISCONTINUATION And Replacement of subjects

## Early Discontinuation of Study Drug

A subject may be discontinued from study treatment at any time if the subject, the investigator, or the Sponsor feels that it is not in the subject’s best interest to continue. The following is a list of possible reasons for study treatment discontinuation:

- Subject withdrawal of consent (or assent)
- Subject is not compliant with study procedures
- Adverse event that in the opinion of the investigator would be in the best interest of the subject to discontinue study treatment
- Protocol violation requiring discontinuation of study treatment
- Lost to follow-up
- Sponsor request for early termination of study
- Positive pregnancy test (females)

If a subject is withdrawn from treatment due to an adverse event, the subject will be followed and treated by the Investigator until the abnormal parameter or symptom has resolved or stabilized.

All subjects who discontinue study treatment should come in for an early discontinuation visit as soon as possible and then should be encouraged to complete all remaining scheduled visits and procedures.

All subjects are free to withdraw from participation at any time, for any reason, specified or unspecified, and without prejudice.

Reasonable attempts will be made by the investigator to provide a reason for subject withdrawals. The reason for the subject’s withdrawal from the study will be specified in the subject’s source documents Refer to Section 10 for early termination procedures.

## Withdrawal of Subjects from the Study

A subject may be withdrawn from the study at any time if the subject, the investigator, or the Sponsor feels that it is not in the subject’s best interest to continue.

All subjects are free to withdraw from participation at any time, for any reason, specified or unspecified, and without prejudice.

Reasonable attempts will be made by the investigator to provide a reason for subject withdrawals.  The reason for the subject’s withdrawal from the study will be specified in the subject’s source documents.  As noted above, subjects who discontinue study treatment early (i.e., they withdraw prior to Visit 2) should have an early discontinuation visit. Refer to Section 10 for early termination procedures.  Subjects who withdraw after Visit 2 but prior to Visit 3 should be encouraged to come in for a final visit (and the procedures to be followed would include those for their next scheduled visit).

## Replacement of Subjects

Subjects who withdraw from the study treatment will not be replaced.

Subjects who withdraw from the study will not be replaced.

# PRotocol Violations

A protocol violation occurs when the subject, investigator, or Sponsor fails to adhere to significant protocol requirements affecting the inclusion, exclusion, subject safety and primary endpoint criteria. Protocol violations for this study include, but are not limited to, the following:

- Failure to meet inclusion/exclusion criteria
- Use of a prohibited concomitant medication

Failure to comply with Good Clinical Practice (GCP) guidelines will also result in a protocol violation. The Sponsor will determine if a protocol violation will result in withdrawal of a subject.

When a protocol violation occurs, it will be discussed with the investigator and a Protocol Violation Form detailing the violation will be generated. This form will be signed by a Sponsor representative and the Investigator. A copy of the form will be filed in the site’s regulatory binder and in the Sponsor’s files.

# DATA SAFETY MONITORING

The Second Affiliated Hospital of Chongqing Medical University Data Safety Monitoring Board (DSMB) will establish a Data Monitoring Committee (DMC) to review data relating to safety and efficacy, to conduct and review interim analyses, and to ensure the continued scientific validity and merit of the study, according to the Second Affiliated Hospital of Chongqing Medical University Data Safety Monitoring Board Operations Manual and a DMC Charter to be established for this protocol. There will be (number of reviews, if any) interim review(s) conducted by the DMC for the purpose of monitoring study conduct and assessing patient safety. Further details regarding the timing and content of the interim reviews is included in the statistical section below.

# STATISTICAL METHODS AND CONSIDERATIONS

Prior to the analysis of the final study data, a detailed Statistical Analysis Plan (SAP) will be written describing all analyses that will be performed. The SAP will contain any modifications to the analysis plan described below.

## Data Sets Analyzed

All eligible patients who are randomized into the study and receive at least one dose of the study drug (the Safety Population) will be included in the safety analysis.

## Demographic and Baseline Characteristics

The following demographic variables at screening will be summarized by dose level: gender, age, BP, WC, Fat %, height and weight.

## Analysis of Primary Endpoint

After application of lira, Physical examination and biochemical tests, OGTT, and EHC tests were performed before treatment, 12 and 24 weeks after treatment.

## Analysis of Secondary Endpoints

If the subjects had serious adverse events within 24 weeks, the application of lira would be stopped. Safety and tolerability data will be summarized by treatment group.

## Interim Analysis

All of the subjects were asked to come back to the outpatient to receive physical examination and biochemical tests, OGTT, and EHC tests at 12 week to assess the effect of lira.

The sample size for this protocol was determined by the patients number of the Second Affiliated Hospital of Chongqing Medical University from January 2017 to December 2019. We planed to enroll 300 subjects for cross-sectional study and 30 subjects for lira administration.

# DATA COLLECTION, RETENTION AND MONITORING

## Data Collection Instruments

The Investigator will prepare and maintain adequate and accurate source documents designed to record all observations and other pertinent data for each subject treated with the study drug.

Study personnel at each site will enter data from source documents corresponding to a subject’s visit into the protocol-specific electronic Case Report Form (eCRF) when the information corresponding to that visit is available. Subjects will not be identified by name in the study database or on any study documents to be collected by the Sponsor (or designee), but will be identified by subject number.

For eCRFs: If a correction is required for an eCRF, the time and date stamps track the person entering or updating eCRF data and creates an electronic audit trail.

The Investigator is responsible for all information collected on subjects enrolled in this study. All data collected during the course of this study must be reviewed and verified for completeness and accuracy by the Investigator. A copy of the CRF will remain at the Investigator’s site at the completion of the study.

## Data Management Procedures

The data will be entered into a validated database. The Data Management group will be responsible for data processing, in accordance with procedural documentation. Database lock will occur once quality assurance procedures have been completed.

All procedures for the handling and analysis of data will be conducted using good computing practices meeting CSE guidelines for the handling and analysis of data for clinical trials.

## Data Quality Control and Reporting

After data have been entered into the study database, a system of computerized data validation checks will be implemented and applied to the database on a regular basis. The study database will be updated in accordance with the resolved queries. All changes to the study database will be documented.

## Archival of Data

The database is safeguarded against unauthorized access by established security procedures; appropriate backup copies of the database and related software files will be maintained.  Databases are backed up by the database administrator in conjunction with any updates or changes to the database.

At critical junctures of the protocol (e.g., production of interim reports and final reports), data for analysis is locked and cleaned per established procedures.

## Availability and Retention of Investigational Records

The Investigator must make study data accessible to the monitor, other authorized representatives of the Sponsor (or designee), IRB/IEC, and Regulatory Agency inspectors upon request. A file for each subject must be maintained that includes the signed Informed Consent, Assent Form and copies of all source documentation related to that subject. The Investigator must ensure the reliability and availability of source documents from which the information on the CRF was derived.

All study documents (patient files, signed informed consent forms, copies of CRFs, Study File Notebook, etc.) must be kept secured for a period of two years following marketing of the investigational product or for two years after centers have been notified that the IND has been discontinued. There may be other circumstances for which the Sponsor is required to maintain study records and, therefore, the Sponsor should be contacted prior to removing study records for any reason.

## Monitoring

Monitoring visits will be conducted by representatives of the Sponsor according to the the ethics committee of the Second Affiliated Hospital of Chongqing Medical University. By signing this protocol, the Investigator grants permission to the Sponsor (or designee), and appropriate regulatory authorities to conduct on-site monitoring and/or auditing of all appropriate study documentation.

## Subject Confidentiality

In order to maintain subject confidentiality, only a subject number will identify all study subjects on CRFs and other documentation submitted to the Sponsor. Additional subject confidentiality issues are covered in the Clinical Study Agreement.

# ADMINISTRATIVE, ETHICAL, REGULATORY CONSIDERATIONS

The study will be conducted according to the Declaration of Helsinki,and approved by the ethics committee of the Second Affiliated Hospital of Chongqing Medical University (2014 Colombo Review No. (72)).

To maintain confidentiality, all laboratory specimens, evaluation forms, reports and other records will be identified by a coded number and initials only. All study records will be kept in a locked file cabinet and code sheets linking a patient’s name to a patient identification number will be stored separately in another locked file cabinet. Clinical information will not be released without written permission of the subject.

## Protocol Amendments

Any amendment to the protocol will be written by Ling Li. Protocol amendments cannot be implemented without prior written IRB/IEC approval except as necessary to eliminate immediate safety hazards to patients. A protocol amendment intended to eliminate an apparent immediate hazard to patients may be implemented immediately, provided the IRBs are notified within five working days.

## Institutional Review Boards and Independent Ethics Committees

The protocol and consent form will be reviewed and approved by the IRB/IEC of each participating center prior to study initiation. Serious adverse experiences regardless of causality will be reported to the IRB/IEC in accordance with the standard operating procedures and policies of the IRB/IEC, and the Investigator will keep the IRB/IEC informed as to the progress of the study. The Investigator will obtain assurance of IRB/IEC compliance with regulations.

Any documents that the IRB/IEC may need to fulfill its responsibilities (such as protocol, protocol amendments, Investigator’s Brochure, consent forms, information concerning patient recruitment, payment or compensation procedures, or other pertinent information) will be submitted to the IRB/IEC. The IRB/IECs written unconditional approval of the study protocol and the informed consent form will be in the possession of the Investigator before the study is initiated. The IRB/IECs unconditional approval statement will be transmitted by the Investigator to the Sponsor prior to the shipment of study supplies to the site. This approval must refer to the study by exact protocol title and number and should identify the documents reviewed and the date of review.

Protocol and/or informed consent modifications or changes may not be initiated without prior written IRB/IEC approval except when necessary to eliminate immediate hazards to the patients or when the change(s) involves only logistical or administrative aspects of the study. Such modifications will be submitted to the IRB/IEC and written verification that the modification was submitted and subsequently approved should be obtained.

The IRB/IEC must be informed of revisions to other documents originally submitted for review; serious and/or unexpected adverse experiences occurring during the study in accordance with the standard operating procedures and policies of the IRB; new information that may affect adversely the safety of the patients of the conduct of the study; an annual update and/or request for re-approval; and when the study has been completed.

## Informed Consent Form

Informed consent will be obtained in accordance with the Declaration of Helsinki and the ethics committee of the Second Affiliated Hospital of Chongqing Medical University.

The Investigator will prepare the informed consent form, assent and HIPAA authorization and provide the documents to the Sponsor or designee for approval prior to submission to the IRB/IEC. The consent form generated by the Investigator must be acceptable to the Sponsor and be approved by the IRB/IEC. The written consent document will embody the elements of informed consent as described in the International Conference on Harmonisation and will also comply with local regulations. The Investigator will send an IRB/IEC-approved copy of the Informed Consent Form to the Sponsor (or designee) for the study file.

A properly executed, written, informed consent will be obtained from each subject prior to entering the subject into the trial. Information should be given in both oral and written form and subjects (or their legal representatives) must be given ample opportunity to inquire about details of the study. If appropriate and required by the local IRB/IEC, assent from the subject will also be obtained. If a subject is unable to sign the informed consent form (ICF) and the HIPAA authorization, a legal representative may sign for the subject. A copy of the signed consent form (and assent) will be given to the subject or legal representative of the subject and the original will be maintained with the subject’s records.

## Publications

The preparation and submittal for publication of manuscripts containing the study results shall be in accordance with a process determined by mutual written agreement among the study Sponsor and participating institutions. The publication or presentation of any study results shall comply with all applicable privacy laws, including, but not limited to, the Health Insurance Portability and Accountability Act of 1996.

## Investigator Responsibilities

By signing the Agreement of Investigator form, the Investigator agrees to:

1. Conduct the study in accordance with the protocol and only make changes after notifying the Sponsor (or designee), except when to protect the safety, rights or welfare of subjects.
2. Personally conduct or supervise the study (or investigation).
3. Ensure that the requirements relating to obtaining informed consent and IRB review and approval meet Chinese guidelines.
4. Report to the Sponsor or designee any AEs that occur in the course of the study, in accordance with the ethics committee of the Second Affiliated Hospital of Chongqing Medical University.
5. Ensure that all associates, colleagues and employees assisting in the conduct of the study are informed about their obligations in meeting the above commitments.
6. Maintain adequate and accurate records in accordance with the ethics committee of the Second Affiliated Hospital of Chongqing Medical University and to make those records available for inspection with the Sponsor (or designee).
7. Ensure that an IRB that complies with the requirements of the ethics committee of the Second Affiliated Hospital of Chongqing Medical University will be responsible for initial and continuing review and approval of the clinical study.
8. Promptly report to the IRB and the Sponsor (or designee) all changes in the research activity and all unanticipated problems involving risks to subjects or others (to include amendments and IND safety reports).
9. Seek IRB approval before any changes are made in the research study, except when necessary to eliminate hazards to the patients/subjects.

APPENDIX 1. Schedule of Study Visits

|  | **Visit 1 (Day/Week/Month #)^a^** | **Visit 2 (Day/Week/Month #)^a^** | **Visit 3 (Day/Week/Month #)^a^** |
| --- | --- | --- | --- |
| Informed Consent | **x** |  |  |
| Medical History | **x** |  |  |
| Complete Physical Exam | **x** |  |  |
| Abbreviated Physical Exam |  | **x** | **x** |
| Height | **x** | **X** | **X** |
| Weight | **x** | **x** | **x** |
| WC |  |  |  |
| FAT% |  |  |  |
| Vital Signs | **x** | **x** | **x** |
| Pharmacokinetics |  | **x** |  |
| Serum Fetuin-B | **x** |  |  |
| Pregnancy Test (Urine or Serum) | **x** |  |  |
| Hematology | **x** |  |  |
| OGTT | **x** |  |  |
| EHC | **x** |  |  |
| Urinalysis | **x** |  |  |
| Dispensing or Administration of Study Drug | **X** | **X** | **X** |
| Counting of Returned Study Drug |  | **X** | **X** |
| Concomitant Medication Review | **X** | **X** | **X** |
| Adverse Experiences |  |  |  |

^a^ ±2 days
